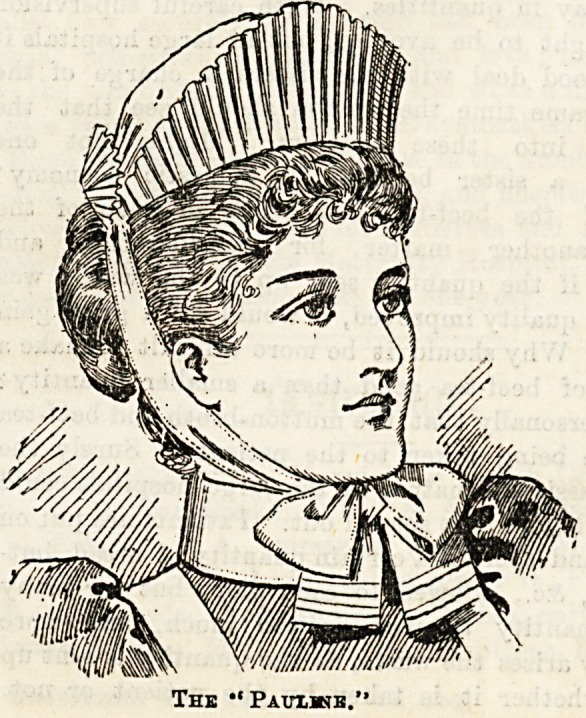# The Hospital Nursing Supplement

**Published:** 1894-10-13

**Authors:** 


"The Hospital" Nursing Mirror.

Being the Extra Nursing Supplement of "The Hospital" Newspaper.

[Contributions for this Supplement should be addressed to the Editor, The Hospital, 428, Strand, London, W.C., and should have the word "Nursing" plainly written in left-hand top corner of the envelope.]

### News from the Nursing World.

### ROYALTY AT LEEDS.

Their Royal Highnesses the Duke and Duchess of York visited Leeds last week for the purpose of opening the hall, library, and medical school of the Yorkshire College of Science, which is affiliated with the Victoria University. The streets were crowded, and a most enthusiastic welcome was accorded to the Royal visitors. The school is connected with the infirmary opened by the Prince of Wales in 1869, and the Duke of York referred to various pleasant visits paid to the city by the Queen and his Royal parents. Some excitement was caused by a man attempting to open the door of the carriage in which the Duke and Duchess of York were driving through the streets. Rumour greatly exaggerated the incident, and it was a relief to the loyal citizens of Leeds and to the nation at large to learn the trivial nature of the occurrence.

### KINDLY ACKNOWLEDGMENT.

A courteous tribute to the value of trained nurses was paid by Dr. William Walter Merry, D.D., in his address at St. Thomas's Hospital the other day. He remarked that their "skill and tenderness had doubled the physician's power and lightened the burden of human suffering." Dr. Merry's humorous allusions to the growing interest taken by the general public in medical science will also be read with interest by nurses who have special opportunities of protesting against the neglected filter and the milk which, like a lodging-house kettle, is "just on" but never actually "boiling." Nurses have opportunities for setting a good example, and giving practical hints in these matters, and a little tact generally ensures their doing so unobtrusively.

### TRAINED NURSES' CLUB.

It is proposed to hold a sale of work in December in aid of the funds of the Midwives' and Trained Nurses' Club, 12, Buckingham Street, Strand. The enlarged premises offer every facility for the undertaking, and contributions of work and useful ornamental wares will be gladly received and acknowledged by the Hon. Secretary. Things suitable for Christmas gifts are certain to find a ready sale, and no doubt the undertaking will be as successful as former ones connected with this excellent club. The large class-room which now belongs to the institution can be hired by the day or hour at a moderate fee, for lectures, lessons, meetings, &c.

### THOUGHTLESS CRITICS.

"I have often wanted to ask why you don't subscribe to our hospital?" asked the intimate friend of a wealthy lady. "Simply because the nursing is so badly organised," returned the latter; "otherwise I would gladly support it, for it seems a most useful institution." Her friend felt puzzled; she had every reason for believing that the nursing department was in all ways satisfactory. "Why do you think it badly organised?" she inquired at last. The wealthy lady looked troubled. "Indeed, I don't speak without knowledge," she said; "I have heard it from a niece of my own. She was there for a month once, and she said she didn't think the place at all well-managed." It transpired that the lightly-spoken condemnation was not only without foundation, but was only half-meant by the young lady, who uttered it in momentary pique on discovering that the profession which she had "taken up" so enthusiastically wasn't a bit what she expected to find it!

### SECTARIANISM IN WORKHOUSES.

"We never inquire into the religion of our officers before they are appointed," remarked a Guardian the other day, and similar testimony is often given incidentally in reports of Board meetings. Whatever the religion of a nurse, she should be free personally to follow the dictates of her own conscience, so long as she attempts no interference with similar liberty for other people. In workhouse infirmaries there is little chance of a nurse having spare time to proselytise amongst her patients. Indeed it is probable that more practical lessons are learnt by the sick poor from daily observance of the devoted, patient nurse, whose duty is performed with cheerful conscientiousness, than by any controversial conversations or pamphlets.

### WINTER WORKERS WANTED.

"Poor little children!" sighs the sentimental visitor. "How can I help them?" asks the practical one. Nobody with a few hours regularly to spare need be at a loss for useful work waits to be done, and needs only goodwill and punctuality to ensure success in it. We have appealed on various occasions for workers, and never in vain, for Miss Coleman, Superintendent of the Hospital for Children, 2, Maida Vale, says she has obtained many good helpers through the columns of The Hospital, and now she needs two more ladies to give their services to the poor children regularly during the winter. One is wanted from 9 to 12.30 two or three times a week, and the other from 3 to 6. They will help the nurses and learn much from the method and skill which the latter exercise. Most of the children require great care, and, alas! some of them are incurable. It is certainly worth while for a young lady who wants "to do something useful" to go and see the little "home and hospital," which will itself appeal more earnestly than any written words can do, for a little seasonable assistance.

### ROYAL NATIONAL PENSION FUND.

The offices of the Royal National Pension Fund for Nurses are now established at the new address, 28, Finsbury Pavement. The entrance door is in West Street, just the width of Milner's shop from the Pavement. If intending visitors will bear these simple directions in mind, they will save themselves trouble and avoid the necessity of asking their way.

### CERTIFICATED BUT NOT TRAINED.

That the popular practice of dressing maid-servants like hospital nurses does not always originate with their employers, is shown in a doctor's letter to the *Lancet* last week. He tells of his surprise at finding a young woman engaged by his wife as a nurse-maid appearing afterwards in the dress of a hospital nurse. On his inquiring where she had been trained, he was informed that she held a "diploma," which proved to be a document signed and sealed by a gentleman whose "course of instruction in midwifery and diseases of women and children" the young woman had attended. After an examination held by the same gentleman this "diploma" had been granted, and it certified the pupil to be "capable of undertaking the duties of a midwife or sick nurse, medical and surgical." It is to be hoped that more particulars will be volunteered on a matter of such importance, and we shall be interested to learn if any of our readers have had their attention called to this last new development in nurses' "diplomas."

### AN INCREASE OF STAFF.

The Chorlton Board of Guardians have decided to add seven probationers to their nursing staff. They are each, according to the local press, to pay a premium of £10 for the first year, and to receive the same sum during the second twelve months. It is anticipated that the introduction of so many probationers will add greatly to the comfort of the sick inmates.

### PROGRESS AT EXETER.

The accommodation for the nursing staff at Exeter Sanatorium is likely to be greatly improved by the contemplated structural additions. The medical officers are said by the local press to have condemned as inadequate the existing arrangements, and the Sanitary Committee have taken the matter in hand, and directed the preparation of suitable plans. A laudable desire to increase the comfort of the nursing staff appears to actuate the Exeter City Council.

### CLASSES AT MANCHESTER.

The students of the Manchester School of Domestic Economy and Cookery received their diplomas on the 5th inst., at a well-attended meeting, presided over by the Lord Mayor. Miss A. Romley Wright, managing trustee and hon. secretary, in reporting on the work of the year, said that she hoped to see dressmaking and millinery added to the subjects of instruction in the near future. She caused some amusement by picturing the gentlemen of the educational department disputing over the number of lessons needed to make a good laundry teacher, remarking that "When England became civilised perhaps they might have women sitting in judgment upon such matters." The courses of Physiology and Hygiene are considered to have been very successful ones.

### QUEEN'S NURSES IN IRELAND.

That good work is done among the Dublin poor by the St. Patrick's Nurses (affiliated with the Queen's Jubilee Institute) needs no further evidence than the fact that 369 patients were nursed by them during the three months ending September 30th, these including 268 new cases, while the number of applications for attendance reached 283, and 5,895 visits were paid by the nurses.

### WELSH NURSES.

There is apparently a very real demand for Welsh-speaking nurses. It is, therefore, urged by some of the residents in the little Principality that suitable candidates should be encouraged to go through the full training necessary to render them the qualified and certificated nurses which Wales desires to employ. Local interest in the work of the district nurse is well maintained at Carnarvon. A grand bazaar, lasting two days, was recently held on behalf of the funds of the association, and the number of visitors ensured the success of the fête. The bazaar was formally opened by Lady Penrhyn, whose interest in the matter of skilled nursing for the sick poor is well known. She spoke with much feeling of the good which had been effected in the neighbourhood by their district nurse and Sir Llewellyn Turner, also bore testimony to the value of the work already accomplished.

### THE NOURISHMENT OF FRENCH BABIES.

What babies are fed on, and what they ought to eat, are often diametrically opposite. The unsuitable diet doled out to infants is the despair of the out-patient physician, the general practitioner, and the district nurse. A novel mode of dealing with the difficulty has just been decreed in France, and it will be interesting to see if the procedure proves efficacious. The Prefects, we are told, are instructed by the Director of Public Health to forbid bringing children up by hand, or allowing those under a year old solid food, unless it is prescribed for them.

### SHORT ITEMS.

The report by the Special Commissioner of the *British Medical Journal* on Stockport Workhouse shows that nothing short of rebuilding can rectify its present overcrowded and insanitary condition, an adequate staff of trained nurses for night and day duty being also required.—The teaching of hygiene in schools was discussed in the section of Sanitary Science and Preventive Medicine at the Sanitary Institute Congress recently held at Liverpool.—The Queen ordered some of the Royal fruit exhibited at the Crystal Palace to be sent on to the patients at University College and St. George's Hospitals.—On the retirement of Nurse Slay, who for twenty years has acted as midwife at Croydon Workhouse Infirmary, she was granted a gratuity equal to two years' salary as a recognition of her long and valuable services to the Board.—At the annual meeting of the Southmolton Sick Nurse Association a satisfactory report of the finances and work was laid before the subscribers; Lady Susan Fortescue was re-elected president.—At Exmouth a carnival is arranged to take place on October 31st in aid of the Town Nurse and Dispensary Funds.—Nurse Woodman and Police-constable Fairley are said to have recently saved a man's life at Weardale by artificial respiration. The man had inhaled gas fumes from a drain, and was unconscious.—The Barton Regis Guardians decide to make some important improvements in the nursing department of their workhouse infirmary. The Duchess of Teck Hospital at Patna is getting on fast, and the roof will soon be completed.—The annual "Hospital Saturday" collections in Cork have been most successful this year, the total recorded being £389, as compared with £360 in 1893. In addition the Cork Steam Packet Company have contributed £111, and various smaller sums have been collected among the employés of other firms.—The twenty-seventh anniversary meeting of the Tottenham Deaconess Institution and Hospital took place on the 6th inst.—A new out-patient department is talked off in connection with the Hospital for Sick Children, Moor Edge; the site and building to be the gift of Lord Armstrong, in memory of the late Lady Armstrong.

### Notes for Nurses on Antiseptic Surgery.

By William Horrocks, M.D., F.R.C.S., Hon. Surgeon to Bradford Infirmary.

### I.—THE DEVELOPMENT OF ANTISEPTIC METHODS.

Perhaps there is no discovery or series of discoveries which have conferred greater benefits, both in saving life and suffering, than the introduction of antiseptics. As all surgical treatment hangs on a good understanding of these methods, I have wished to bring before you in a simple way some of the fundamental principles on which the treatment is founded. I think this more important as you all have constantly to treat wounds, and may by mistakes bring on serious results, as a single error may destroy all the good which ought to follow careful application of antiseptic rules.

### Chance or Luck.

First, I would endeavour to free your minds from the factor of chance or luck in wound treatment. We get two patients operated on by the same surgeon, on the same day, apparently both in a similar state of health, yet in one the wound heals without fever, while in the other the wound gapes, suppurates, and finally heals only after weeks of trouble. We must all have seen such cases, and felt inclined to say how little the art of wound healing is understood, and how much is a matter of chance or luck. Now I have always fought this feeling, as it necessarily leads to carelessness; for if wounds heal or suppurate for some unknown reason, why not let things drift?

### Surgery Thirty Years Ago.

This want of certainty was, in fact, the condition of surgery thirty years ago. Operations were performed without any certainty as to the ultimate results. Some wounds healed up without the formation of matter, the vast majority healed after suppuration by granulations. At intervals erysipelas or pyæmia carried off case after case. It is to explain how we have advanced to greater precision that I wish to speak.

First we must consider a subject with which you are all more or less familiar, and which will serve to explain some fundamental principles.

### Yeast.

We all know the substance called barm, or yeast. It is a brownish, crumbling mass, which when placed in water forms a frothy or yeasty fluid. If this yeast fluid is placed in dough, which is kept in a warm place, the frothy yeast penetrates the dough and causes it to rise. In other words the yeast, which is a plant, grows in the flour, changing the flour and producing bubbles of gas which permeate the dough and make the bread full of small holes, or, as we say, light and well risen.

In this experiment, which is familiar to all, I ask you to notice certain points:—

1. The barm or yeast: (i.) Must be living or good, before the leavening takes place; (ii.) The yeast actually increases in amount, the amount of growth depending on the amount of food and the length of time; (iii.) That in growing it produces certain products.

2. That the yeast will not grow unless placed in a suitable medium. The plant must have a soil to grow in if growth is to go on to any considerable extent. If yeast is placed in water only, the growth soon stops.

3. That certain conditions are necessary: (i) A certain temperature favours the growth, and if the temperature is lowered much the growth is arrested, if lowered still further the yeast may be killed. If the temperature is raised too high the yeast may be killed; (ii) moisture favours the growth of the plant.

Hence in rising of dough we have present: (1) A ferment capable of indefinite growth; (2) a pabulum or food in the flour; (3) favourable external conditions as regards growth: (*a*) heat; (*b*) moisture.

### Proved by Pasteur.

It was the study of the growth of the yeast plant by Pasteur which was the first step towards the solution of the difficulties of antiseptic surgery. Pasteur proved that yeast was a plant, which was capable of indefinite growth in a suitable material under favourable circumstances. That it was killed by extremes of heat or cold. He proved that to produce fermentation three things were necessary, (1) the ferment; (2) the material to grow it in; (3) suitable surroundings.

### Pasteur and Lister.

It was Pasteur's discoveries which caused Lister to begin his experiments. His original idea was that there existed almost universally a material which contaminated wounds. That this material or poison floated in the air, polluted water, instruments, sponges, and so gained access to wounds.

### Sepsis of Wounds.

He considered this poison or sepsis of wounds was to be prevented in two ways by the covering up or protection of the wound surface to hinder the material from falling on the wound, and by the destruction of the material by certain agents. He called this poison sepsis (σεπτω, to rot) and his method of destroying the poison was called antiseptic.

### Lister's Theory.

Lister's theory assumes, that this poison, like yeast, is a living material, which is capable of indefinite growth in a suitable medium, and under suitable conditions. In other words he requires the same three essentials as are required for the growth of the yeast plant. (1) The ferment or septic material. (2) The food for it to grow in. (3) The suitable surroundings.

### Foundation of His Theory.

You will ask on what facts did the theory rest. Everyone must have noticed, when a beam of light passes through a chink into a dark room, the air, which seems so clear in the sunlight, is seen to be loaded with particles, which float about in countless numbers. These particles settle on surfaces and make what is technically known as dust. Lister then said that the poison of wounds floated in the air with the dust.

### His Experiments.

To prove this he used two kinds of experiments. He took test tubes partly filled with some material liable to decompose, as beef tea, and boiled them to destroy the ferment present in them. The tops of some tubes were left open, the tops of others were filled with plugs of cotton wool, so that the dust was unable to find its way into the fluid. He found that the unprotected fluids soon became putrid, but the protected tubes kept good for an indefinite time. He now went a step further: he found if the vessel had a wide mouth, so that more dust dropped, it went bad sooner than if it had narrow mouth. He found that the dust would not pass a bend in the tube. If he took a flask with a V-shaped bend in its neck and placed fluid in the flask and in the V bend, that the fluid exposed to dust in the V bend went bad, while the fluid in the flask remained sweet. In favour of this view were two facts well known in surgery, which will be referred to later.

### Minor Appointments.

Fever Hospital, Stockton-on-Tees.—Miss Mary Seal has been made Sister at this hospital. She was trained at Monsall Hospital, and was Staff Nurse there, and afterwards Charge Nurse at the Southport Borough Hospital, of which she took charge during the Matron's absence. Miss Seal's testimonials are excellent, and we wish her every success.

### New South Wales.

(By a Sydney Correspondent.)

At a meeting of the directors of the Sydney Hospital held towards the latter end of July, two additional resident medical officers, Dr. Mackinnon and Dr. Veech, were appointed. This places the residential staff on the same footing as that of the Prince Alfred Hospital, viz., two house surgeons, and two house physicians, at a salary of £50 per annum, one senior resident medical officer, at a salary of £100 per annum, and the medical superintendent at a salary of £360 per annum. At the Prince Alfred the salaries differ since they are as above, but less ten per cent. The medical superintendent getting £400 less ten per cent., which makes his income the same as that given at the Sydney Hospital. As the finances of the Prince Alfred are now in a better condition than they have been for years, the salaries might well be raised to their former standard. At both these large Sydney hospitals, the residential staff is entirely composed of graduates of the Sydney University medical school, excepting the medical superintendent of the Sydney, who is a Melbourne graduate.

The new buildings for the Sydney Hospital are rapidly approaching completion and are to be opened shortly.

The circle of trained nurses seems to be overstocked, or there are a number of nurses who wish to obtain the matronships of smaller hospitals, for there were 26 applications for the matronship of the Western Suburbs Cottage Hospital. Of these, four were selected by a special committee, three being old Prince Alfred nurses, Miss Henson being finally appointed. She was lately a nurse in charge at the P.A.H. where she was trained.

The position of Matron to the children's hospital at Glebe Point has recently changed hands. Miss Matthews resigned, Miss Hilda Player, who was Head Sister at the hospital, being selected out of a number of applicants.

Three of the doctors connected with the children's hospital, or rather, with the diphtheria ward, lately developed that disease, all within a few weeks of each other. Happily, the attacks proved to be mild ones, and the medical gentlemen are all on duty again. Doubts were expressed as to the nature of the throat affection, but cultures made, and Loeffler's bacillus demonstrated, set the matter at rest.

Towards the latter end of last year a meeting was held in the Town Hall, Sydney, and presided over by the Mayor, for the purpose of initiating a Hospital Saturday Fund. A large and representative committee was appointed, and April 28th chosen for Sydney's first Hospital Saturday, 400 women, acting as collectors, took possession of all vantage spots in the city. A meeting was held in the Town Hall on Augus 3rd, presided over by His Excellency the Governor, Sir Robert Duff. The Secretary of the Hospital Saturday Committee, Mr. C. H. Starkey, read the report, and gave out the following grants :—

| | | | |
|---|---|---|---|
| Sydney Hospital ... ... ... ... | £553 | 16 | 11 |
| St. Vincent's Hospital ... ... ... | 320 | 15 | 4 |
| Hospital for Sick Children ... ... ... | 311 | 10 | 9 |
| The Carrington Hospital (Convalescent)... | 138 | 9 | 3 |
| Balmain Cottage Hospital ... ... ... | 138 | 9 | 3 |
| North Shore Hospital ... ... ... | 138 | 9 | 3 |
| Parramatta Hospital... ... ... ... | 138 | 9 | 3 |
| Lewisham Hospital ... ... ... ... | 60 | 0 | 0 |
| Total ... ... ... | £1,800 | 0 | 0 |

The ordinary expenditure in connection with the movement was £49 11s. 2d.; payment for goods required to make the collection increased the total outlay to a little over £100.

It will be noticed in the distribution that the Prince Alfred Hospital is not mentioned, the board of directors generously wished not to participate this year, since that hospital had a special collection last in a self-denial week, whereby it was enriched by £4,000.

Recognising the growing importance of the Hospital Saturday movement, it was registered under the Companies Act as an association for charitable purposes. The articles of association were agreed to at the meeting.

### The Countess of Dufferin's Fund.

This fund owes its existence, as we read in the annual report, to the interest displayed by the Queen, Empress of India, in the condition of the women and children of India; its success being due to the able manner in which the scheme was inaugurated in that country by the Countess of Dufferin and Ava.

The association aims at supplying medical relief by establishing hospitals, dispensaries, and wards, under the superintendence of qualified women doctors; and also provides for educating women in India as doctors, hospital assistants, nurses, and midwives.

### Queen-Empress' Medals.

A gold medal will be available every year, which will be awarded by the university authorities at Calcutta, Lahore, Madras, and Bombay respectively to the students who pass certain medical examinations with credit.

### Viceroy's Medals.

Each year three silver medals will be available for female students of the hospital assistant class at Calcutta, Lahore, and Agra, and at the latter a bronze medal is awarded annually by the president.

At Madras a silver medal is given every year to female students who qualify as medical practitioners.

Ten silver and four bronze medals, which were presented to the association by the Marquis of Dufferin and Ava, were allotted for competition in Bengal, Calcutta, and Madras.

A number of scholarships, of which particulars are given in the printed annual report, show that substantial aid is offered to, amongst others, those native ladies willing to come to England to complete their medical education.

The prognostication that the association would materially suffer by Lady Dufferin's departure from India happily proved futile, probably because, as Sir P. Hutchins remarked, "those who indulged in these forebodings entirely failed to realise how securely the scheme had been founded on the eternal principles of humanity and love; how widespread was the want which it supplied, and what a firm hold it had at once taken on the affections and sympathies of all classes." So there has been a steady increase in the number of qualified medical women, assistant surgeons, and hospital assistants.

There is a training home for midwives and nurses in connection with the Dufferin Maternity Hospital, Rangoon, and the course of instruction lasts for one year.

### Marriage.

On October 6th, at St. Marylebone Parish Church, by the Rev. E. Gupper Banks, D.D., M.A., assisted by the Rev. D. G. Douglas, M.A., curate of Westward, Wigton, Charles Imray Kirton, M.B., Lond., eldest son of W. F. Kirton, Esq., J.P., of Grenada, West Indies, to Lilian, only daughter of Edward Woakes, M.D., of 78, Harley Street, London.

For Everybody's Opinion, Dress and Uniforms, &c., see p. xv et. seq.

### Everybody's Opinion.

[Correspondence on all subjects is invited, but we cannot in any way be responsible for the opinions expressed by our correspondents. No communications can be entertained if the name and address of the correspondent is not given, or unless one side of the paper only be written on.]

### THE COST OF PROVISIONS.

"A Provincial Matron" writes: I have read with interest your article on the "Cost of Provisions," and I think a system of accounting for board for each inmate daily in all hospitals might be adopted without much trouble. Our hospital is only a small one—21 beds, resident staff, matron, two nurses, and two servants. Of course, there is a daily record kept of the patients; we have that on the diet-sheet; then there is daily in the hospital the same number of officers, viz., five. Our accounts are made up and paid quarterly; at the end of the quarter I make out, with the help of the diet-sheet, the number of patients in the hospital each day during the three [months, adding on to the daily number the resident staff of five. I then divide the result by the number of days in the quarter (usually 91 or 92) which gives me the average number of inmates in the house each day. I make an account of all the provisions bought, including wine, beer, and spirits, add them up, and divide the result by the average number of inmates per diem—this gives the cost per quarter of each individual. For instance, during the quarter, ending June 30th, we had a daily average number of 24 inmates. The cost of provisions for this quarter amounted to £84 16s.; that sum divided by 24, gives a cost of £3 10s. 8d. per quarter, or £14 2s. 8d. per annum per head, patients and staff included. I find each qua rter come to very nearly the same. It would be difficult in a small hospital, I think, to separate the accounts of provisions for staff from those of patients. The patients are provided with everything (they may have fresh eggs only brought in), the diet is liberal and good—meat, legs of mutton, ribs of beef (occasionally rabbits), we are charged 8d. a pound for. The bread, seconds, is good; quantity, unlimited; the butter best Danish, 1 ounce a-day for patients, 8 ounces per week for staff. Milk, 1 pint to 1½ pints for patients on ordinary diet; soup, or broth, according to diet; milk puddings. As a probationer in a large London hospital, it always struck me what an unneceesary amount of waste there was, and I resolved, if ever I became a matron myself, that I would take a special interest in the domestic economy of the institution; and that I would, if possible, improve the quality of the food usually set before the sick in hospitals. I have seen the ward-maids throw away quarts of milk and beef-tea—to empty the cans, I suppose, for the fresh supplies; bread, also, was thrown away in quantities. With careful supervision this, I think, ought to be avoided, but in large hospitals it would rest a good deal with the sisters in charge of the wards; at the same time the matron should see that the sisters looked into these matters. Might not one qualification in a sister be that of domestic economy? The quality of the beef-tea and broth in some of the hospitals is another matter for consideration and improvement; if the quantity sent up to the wards was reduced and the quality improved, it would be a great gain to the patients. Why should it be more difficult to make a large quantity of beef-tea good than a smaller quantity? I always see personally that the mutton-broth and beef-tea are good before being taken to the patients. Surely the housekeeper or assistant-matron in the large hospitals could test the quality before it is served out. Patients are put on different diets, and there is a certain quantity of bread, butter, meat, milk, &c., allowed to each diet; but for many invalids the quantity allowed is too much, and here I suppose partly arises the waste, as the quantity is sent up to the wards whether it is taken by the patient or not. These are the opportunities for the sister to exercise her judgment; not in altering the diets, but in noting down that a less quantity would be sufficient. The amount of waste that goes on in some hospitals will easily account for the much higher cost of provisions in certain institutions as compared to others. Probably a great deal of it goes on in the kitchen as well as in the wards—bones, scraps, and trimmings are not made the best of; soup well made and thickened with peas, and flavoured with vegetables, is an acceptable change for supper instead of milk or beef-tea to the more convalescent patients (especially the men); this reduces the milk account at very little cost. Stewed fruit occasionally instead of continual rice or tapioca is no more expensive when fruit is cheap, and quite suitable for some patients; in fact, there are many ways of varying the monotonous diet without much trouble and no additional cost. The chief way of reducing the cost of provisions is to see that no waste is allowed, a difficult, but possible, task amongst a large staff of servants. I was at one of the London hospitals where the cost per bed for provisions (according to your table) was over £30 per annum, and I can truly say that the patients' diet here, at a cost of not more than £15 per bed, is better and more palatable, both as regards meat, beef-tea, bread, butter, puddings, &c.

### NURSING IN PARIS.

"A Paris Nurse" writes: I am pleased to see that at last the circumstances of the Levick Nursing Institution are made public. There are many nurses both in England and on the Continent who left good positions in hospitals to come to the Levick Institution for a year's Continental experience, and most of them have been unable to get their full salary paid to them. I myself am one of the victims, but my loss, about one hundred francs, is comparativ ely small. With "Levick Nurse," I indeed hope that this correspondence will serve as a warning to other nurses who are thinking of joining.

### Where to Go.

Exhibition of Paris Salons Paintings, Continental Gallery, 157, Bond Street.

This is the first of the autumn exhibitions to open its doors to the public, and we always hail its openiug with much pleasure. Neither too large nor too small, the Continental is a delightiul picture gallery. Though there are several canvases of especial interest this year, perhaps there is no one which is especially striking or remarkable. Among the figure pieces, a treatment of the nude stands out prominently; this is No. 115, "Nymph of the Chase," painted by Joseph Wencker, a pupil of Gérome's. It is the life-size representation of a beautiful woman standing in solitude by a woodland stream. The flesh tints are beyond criticism, the face alone detracting from the general excellence of the scheme. The features are a little over-painted, when compared with the simplicity of the figure, wherein the beauty of the picture lies. "Pierriette and Pierrot" (No. 44), by M. Felin, is an attractive study of a young girl lying on a bed asleep, her attitude suggestive of deep repose, curled up by her side being Pierrot, the cat. The religious studies are pretty well confined to two, neither of which are good specimens, except as examples of the modern treatment of holy subjects, which is rarely worthy of much study. "Gyp," the racy smart novel writer, is represented on these walls with an oil sketch called "Bébé." This versatile artist's brush is not up to his pen, by any means, but the picture has a certain pretty daintyness about it. Really clever is No. 88, a "Study," by Planels-Ricardo, showing us a girl's figure bathed in a bright light, which floods the whole picture. Miss Helen Carlisle is at her best in three canvases, all of which are charmingly executed portraits of women. The best portrait, however, in this collection is of M. de Blowitz, the Paris correspondent of the *Times*, which is very strongly painted in by Benjamin Constant. There is also some good landscape work in the collection, much of which is deserving of a more particularised mention.

### Dress and Uniforms.

By a Matron and Superintendent of Nurses.

### III.—INDOOR.

The most important part of the costume, however, is the cap, whose shape is legion. Every institution has its own ideas on the point, and all wear it somewhat differently. Some caps are very pretty, others again are very ugly. Nothing makes or mars a nurse so much as the way she puts on her cap, and the greatest care is necessary in making them up when they come from the wash. The cap should always be fresh and spotless in appearance, and, with care, should last clean a week. Among the most becoming of caps is the neat little Marie Stuart shape trimmed with two rows of gophered frilling or lace, and finished off with a compact little bow under the chin. This cap requires very great care in making up, or the shape is apt to be lost. Long streamers, though graceful looking, are rather in the way and therefore not to be recommended. A very nice cap is of an oval mob shape, with two rows of rather wide Coventry frilling round the edge and fitting like a coronet to the head. The Sister Dora, though not so smart looking as the Marie Stuart shape, is equally pretty and useful. It fits the head closely, somewhat like a night-cap, and is made of fine white muslin, and has strings of the same, which can be tied or not, at the discretion of the wearer, under the chin. One of the neatest and most useful caps is composed simply of a square of cambric edged with Valenciennes lace which is folded triangularly, the point coming in front and the two ends tied under the hair behind. The prettiest cap, however, fails in its intention if the hair beneath is not kept faultlessly neat and well arranged. It is an index to the whole character, and nurses are earnestly entreated to pay great attention to this most important particular. The question of shoes is one that can only be answered by experience; some nurses find one sort suit their feet best, and others another. Beyond censuring a nurse's wear-high heels and pointed toes, the particular class of shoe must be left to the judgment of the wearer. In our opinion a plain Court, short, with rounded toes, and a square military heel, will be found as comfortable as any, and always looks well.

The "Angelus."

There can be no doubt that when employed in the active discharge of her duties a nurse should always appear in uniform. We regret, however, to observe that there exists a disposition on the part of many nurses engaged in private nursing to disregard this rule. Now this is a pity for more reasons than one. The majority of women look their best in uniform; it gives an air of refinement to the most homely wearer, while to the already refined it still further enhances their charm. A soldier would never think of appearing on parade without his uniform; and the same obligation attaches in our opinion to a nurse when she is on duty. Besides a nurse occupies an official position when she is in attendance on the sick, which it is desirable, both for herself and the patient's friends should be fully recognised. We cannot think therefore that a coloured blouse or a coat and vest of startling pattern are altogether in keeping with that idea of a "ministering angel" with which we like to associate our nurses. The moral effect of uniform is immense, its appearance alone gives the wearer an authority which ordinary garments are powerless to confer. We, therefore, urge on every nurse the propriety of wearing uniform when engaged at a case, unless for some adequate reason she is requested to do otherwise by the patient's friends. There are so many pretty uniforms to be had at a reasonable cost that we wonder at any nurse who studies her appearance hesitating to procure one. Among the most energetic purveyors of this class of goods we notice Messrs. Garrould, of the Edgware Road, who have made a speciality of nurses' uniforms with very great success. Their cloaks are particularly nice, that known as the "Angelus," of which we give an illustration, being exceptionally neat and pretty. The price, too, is very moderate, ranging as it does from 21s. upwards, and it can be had in all colours. A charming little straw bonnet trimmed with velvet the same colour, and a white border, costs 10s. 6d., and will be found becoming to most faces. Caps there are in an infinite variety of shapes, and we admire them almost as much for the excellent arrange-

The New "Sister Dora."

The "Belmont."

The "Pauline."

ment by which they untie and become flat for washing as for their style. We give illustrations of three, which cannot fail to commend themselves to the taste of our readers. The prices, which are very reasonable, place them within the reach of all. The new "Sister Dora" cap is simplicity itself, and only costs 1s. The "Belmont" and the "Pauline" are an elaboration on a somewhat similar principle, and are both pretty and workmanlike. Their cost respectively is 1s. 3d. and 1s. 6d. The aprons made by Messrs. Garrould at 2s. 6d. each are good value for the money, and can be had now in linen as well as in cotton.

### The Book World for Women and Nurses.

[We invite Correspondence, Criticism, Enquiries, and Notes on Books likely to interest Women and Nurses. Address, Editor, The Hospital (Nurses' Book World), 428, Strand, W.C.]

### MAGAZINES OF THE MONTH.

The Pall Mall Magazine contains such excellent matter for this month that it is hard to mention one article beyond another in criticising. Perhaps the most striking among these contents is a sketch which Mrs. Elizabeth R. Pennell entitles "Out of Our Window," and the illustrations to which, though bearing no signature, are quite undoubtedly her husband's work. Which is more charming the letter-press or the pictures it is difficult to say, both being the outcome of the highest art: the art that is, that not only sees beauty in things around and idealises it, but also represents in the poetical manner shown in this sketch. Mr. Pennell's drawings ought each to be cut out and framed so good are they, and also their reproductions. But, as we have said, the excellence of the magazine does not rest alone on this one article. Lord Roberts adds another chapter to "The Rise of Wellington," Rider Haggard, Walter Besant, and Lady Lindsay's contributions maintain the high standard ever shown us in these pages. Mrs. Clifford's little story is remarkably clever, fresh, and interesting.

The new series of The English Illustrated Magazine commences with the October number just issued, among the contents being a story by Gilbert Parker, "A Moorland Idyll," by Grant Allan, and an article by Colonel Howard Vincent, C.B., M.P. This "Measuring Identification of Criminals," on which this great criminal authority bases his remarks, is getting—may we say it—just a little stale. So many papers have taken it up, that one feels almost as if one ought to look upon one's fellow creatures as criminals all round, if one only had the "measuring" arrangements of which the French boast so much, whereby crime certainly seems pretty surely brought home to one, through the instrumentality of the inch tape. What seems to us to be the most striking feature in the new series of our highly-esteemed contemporary is the charming frontispiece which commences it. This is quite beautiful and suggestive, and being reproduced in colours, adds a fresh interest to the magazine.

The Sunday at Home sustains its standard of sound and wholesome literature. Here in the October number we find chapters xxiii. to xxvi. of "The Mystery of Alton Grange," "Woman's Work in the Christian Sphere," with illustrations of the pioneers among us. "A Ride to Little Tibet," by the Rev. J. Hobson, and "The Tauist Priest" are both interesting chapters.

The Cornhill Magazine for October continues its chapters in the two serial stories, and gives us some excellent reading besides. "The Modern Woman" in "Character Notes" is the best which has yet appeared on the subject. "Humours of Heraldry" tells us a good deal on a subject of which, so far, we have seen the graver side. In the reign of Henry VIII. family arms began to assume a more complicated and elaborate character, insomuch that some of them have been compared to a garrison well stocked with fish, flesh, and fowl. The adoption of a florid style of armory was followed, as Boutell says, by the substitution of pictorial representations, often of a most frivolous and unintelligible description, for the simpler and dignified insignia of true heraldry. One family is described as having a grant of arms made in 1760, which, with thirteen other figures, included the representation of a book duly clasped and ornamented, having on it a silver penny, upon which was written the Lord's Prayer, while above the book hovered a dove with a crowquill in its beak.

### Novelties for Nurses.

Autumn brings with it fresh novelties in clothing and dress materials, and frosty mornings warn us that we can no longer delay the purchase of warmer garments. We have received a packet of samples from Messrs. Lutas Leathley and Co. (department 46), the Dress Warehouse, Armley, Leeds, which, for excellence of quality and reasonableness of price, we have not often seen equalled. At this season of the year goods such as we are describing are eagerly sought after, when an intermediate garment, not too expensive, but pretty and serviceable, is required. This firm is especially noted for its cloths and serges, the "Wylwyrwell" cloth which they manufacture being quite the cheapest and prettiest for the money we have ever seen. It can be procured in all shades and colours, from delicate drab to navy blue, with some particularly pretty art shades in blue and green, for the astonishingly low figure of 10s. 6d. the dress length. Rather more expensive, but charming in texture and appearance, are the "Shetland" hopsacks at 2s. 9d. the yard. We can also recommend very highly the "Ideal" cloths and serges at 15s. the piece. The "Zuper" cloth has a lovely smooth satin finish and is warranted not to wear rough. For a "smart" dress nothing could possibly look better. We would draw particular attention to the fact that Messrs. Leathley and Co. pay carriage on all parcels, that patterns are sent post free, and do not require to be returned, which latter consideration is one that will recommend itself to those of our readers whose time is fully occupied and leisure scanty.

### Notes and Queries.

### Queries.

(11) *Nurse Dolls.*—On what terms can these be borrowed to exhibit for a charity?—*Matron.*

(12) *Convalescent.*—To whom should a primary application for assistance from The Hospital Convalescent Fund be made?—*Tired Nurse.*

(13) *Infants.*—I should be glad of an opinion respecting a plan for opening a home for sickly infants. I should only keep them until they were four, and then pass them into other homes. Payment would be according to means, from 2s. 6d. to 5s. per week.—*Inquirer.*

(14) *Monthly Nursing.*—Can you tell me of any hospital that gives this training free to women with no previous knowledge of nursing?—T. P. C.

(15) *Practical Talks.*—Will you kindly send me the address of Miss Isabel Dodgson, whom you mentioned recenfly in The Hospital?—*Nurse E.*

### Answers.

(11) *Nurse Dolls (Matron).*—The Hospital Collection of Nurse Dolls has always been lent without any charge, to assist the funds of institutions for the sick, nursing associations, &c. Beyond paying the carriage of these small visitors, no expense whatever is incurred by the borrower.

(12) *Convalescent (Tired Nurse).*—Address Hon. Secretary, The Hospital Convalescent Fund, 428, Strand, London, W.C.

(13) *Infants (Inquirer).*—We cannot give an opinion without more knowledge of the circumstances. When children do not require hospital treatment, but only proper feeding and good air, it seems to answer well to board them out with respectable cottagers.

(14) *Monthly Nursing (T. P. C.).*—We know of none.

(15) *Practical Talks (Nurse E.).*—If you send a letter to Miss I. Dodgson, care of the Editor of The Hospital, it shall be forwarded. You must always sign your full name and address.

## Figures and Tables

**Figure f1:**
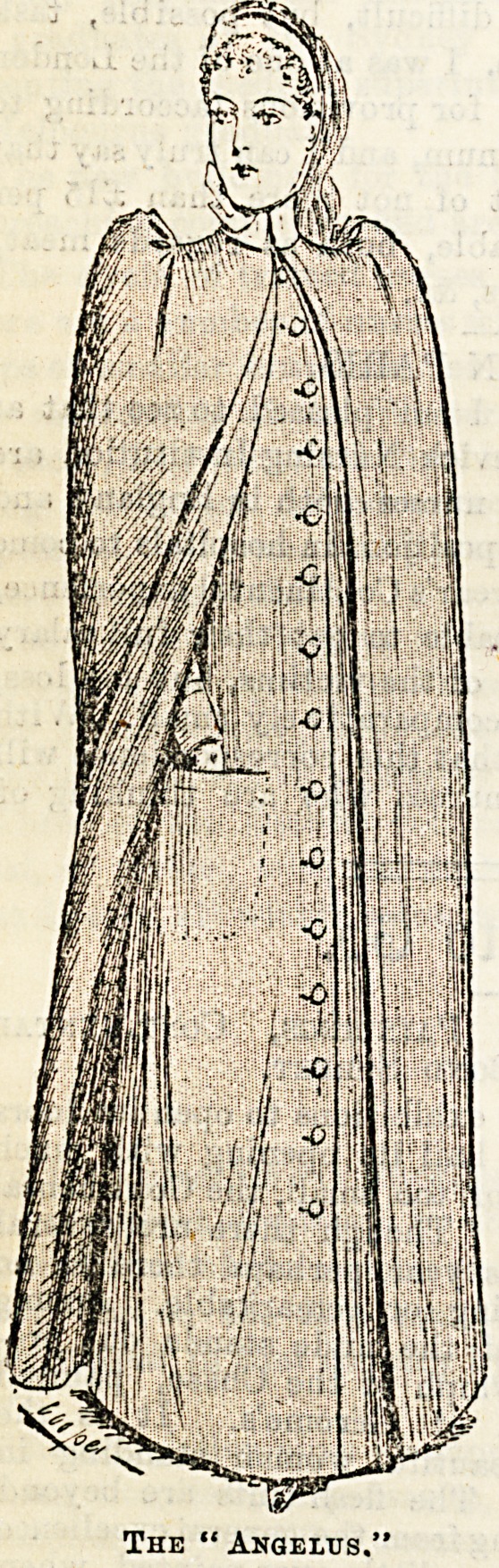


**Figure f2:**
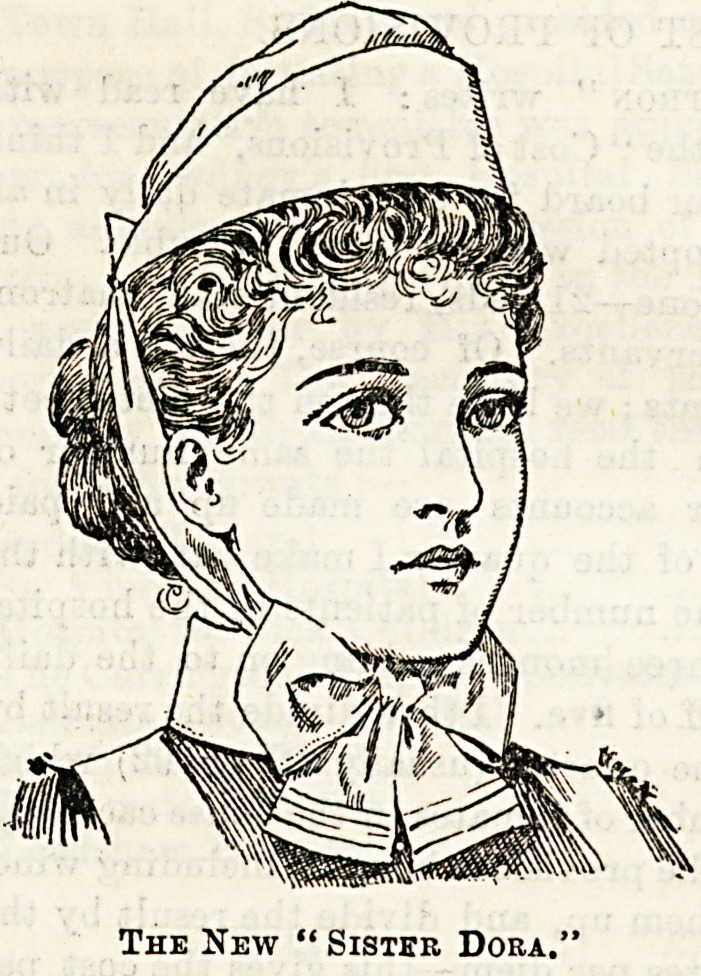


**Figure f3:**
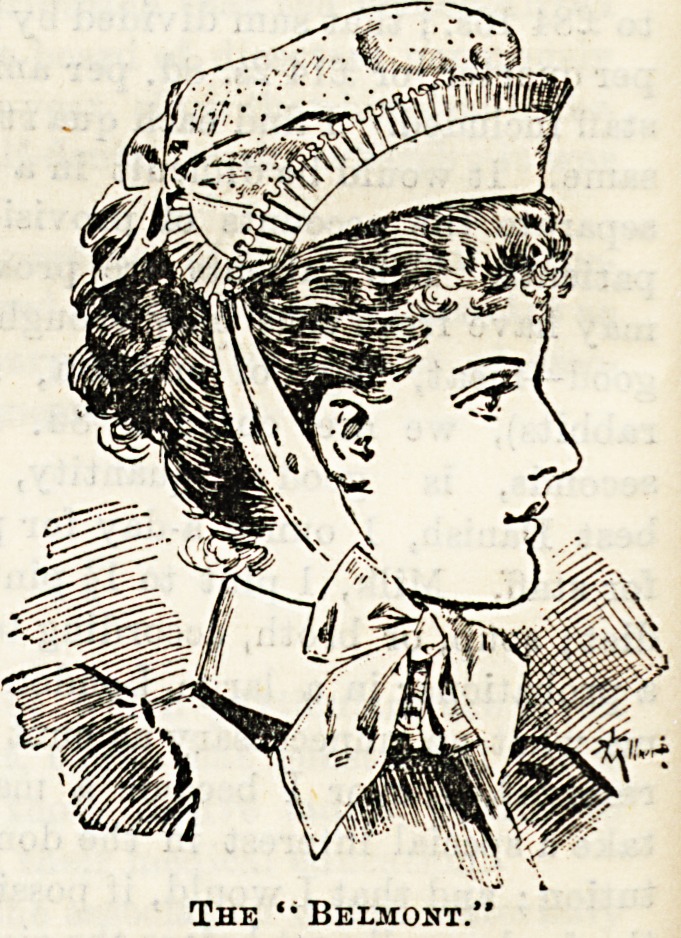


**Figure f4:**